# Facial affect recognition in context in adults with and without TBI

**DOI:** 10.3389/fpsyg.2023.1111686

**Published:** 2023-08-14

**Authors:** Lyn S. Turkstra, Sam Hosseini-Moghaddam, Sophie Wohltjen, Sara V. Nurre, Bilge Mutlu, Melissa C. Duff

**Affiliations:** ^1^Faculty of Health Sciences, McMaster University, Hamilton, ON, Canada; ^2^University of Toronto Schools, Toronto, ON, Canada; ^3^Department of Computer Sciences, University of Wisconsin-Madison, Madison, WI, United States; ^4^American Speech-Language-Hearing Association, Rockville, MD, United States; ^5^Department of Hearing and Speech Sciences, Vanderbilt University, Nashville, TN, United States

**Keywords:** traumatic brain injury, speech-language pathology, emotion recognition, affect recognition, social cognition, facial expression, eye-tracking, communication

## Abstract

**Introduction:**

Several studies have reported impaired emotion recognition in adults with traumatic brain injury (TBI), but studies have two major design features that limit application of results to real-world contexts: (1) participants choose from among lists of basic emotions, rather than generating emotion labels, and (2) images are typically presented in isolation rather than in context. To address these limitations, we created an open-labeling task with faces shown alone or in real-life scenes, to more closely approximate how adults with TBI label facial emotions beyond the lab.

**Methods:**

Participants were 55 adults (29 female) with moderate to severe TBI and 55 uninjured comparison peers, individually matched for race, sex, and age. Participants viewed 60 photographs of faces, either alone or in the pictured person’s real-life context, and were asked what that person was feeling. We calculated the percent of responses that were standard forced-choice-task options, and also used sentiment intensity analysis to compare verbal responses between the two groups. We tracked eye movements for a subset of participants, to explore whether gaze duration or number of fixations helped explain any group differences in labels.

**Results:**

Over 50% of responses in both groups were words other than basic emotions on standard affect tasks, highlighting the importance of eliciting open-ended responses. Valence of labels by participants with TBI was attenuated relative to valence of Comparison group labels, i.e., TBI group responses were less positive to positive images and the same was true for negative images, although the TBI group responses had higher lexical diversity. There were no significant differences in gaze duration or number of fixations between groups.

**Discussion:**

Results revealed qualitative differences in affect labels between adults with and without TBI that would not have emerged on standard forced-choice tasks. Verbal differences did not appear to be attributable to differences in gaze patterns, leaving open the question of mechanisms of atypical affect processing in adults with TBI.

## 1. Introduction

Traumatic brain injury (TBI) is a leading cause of death and disability worldwide, with an estimated annual incidence of 69 million (Dewan et al., 2018). While individuals with milder injuries may recover pre-injury function, more than 20% of survivors will live with chronic impairments in sensorimotor and cognitive functions (Masel and DeWitt, 2010; Blennow et al., 2016).

Impairments in social functioning are among the most pervasive and disabling consequences of TBI, affecting up to 70% of all survivors (Benedictus et al., 2010). Indeed, adults with TBI report fewer regular social contacts than their uninjured peers, less social participation, and more isolation (Stocchetti and Zanier, 2016), all of which have negative effects on employment, health, and quality of life (McLean et al., 2014). Contributing to negative social outcomes are impairments is the ability to recognize emotions from others’ faces, voices, and body postures (Babbage et al., 2011; Zupan and Neumann, 2014; Byom et al., 2019; Neumann and Zupan, 2019; Turkstra et al., 2020). These impairments are particularly prevalent among those with moderate or severe TBI (Murphy et al., 2021), although they have been reported in some individuals with mild TBI (concussion) as well (Theadom et al., 2019). Impairments in emotion recognition can have a significant negative effect on broader social outcomes (Genova et al., 2017; Milders, 2018; Rigon et al., 2018; Rosenberg et al., 2018; Binder et al., 2019; Sherer et al., 2022), may play a role in rehabilitation of non-emotional functions (Spikman et al., 2013), and may be remediable (Cassel et al., 2019; Vallat-Azouvi et al., 2019). As a result, assessment of the ability to recognize emotions has been recommended for clinical management of patients with TBI (Togher et al., 2023).

While assessment of affect recognition is important, existing clinical and research assessment tools have several limitations. First, stimuli are typically faces presented in isolation. As Aviezer et al. (2017) observed, the notion that we can infer others’ emotions from their faces alone “is deeply ingrained in lay intuition, popular culture and scientific thought” (p. 47). Indeed, faces alone do not capture the rich visual, cultural, and social contexts in which we interpret affective displays in everyday life (Barrett et al., 2011; van Kleef et al., 2016). Studies over the past three decades have shown that recognition and interpretation of emotions is highly influenced by—and in many cases dependent on—context cues (Carroll and Russell, 1996; de Gelder et al., 2006; Righart and de Gelder, 2008a,b; Wieser and Brosch, 2012; Schwarz et al., 2013; Nelson and Mondloch, 2019). Context cues can be within the expresser (e.g., body posture) (Aviezer et al., 2012), within the scene (e.g., background cues to events) (de Gelder et al., 2006), or within the observer (e.g., the person’s mood) (Aviezer et al., 2017), and may override facial affect cues in some contexts (Kayyal et al., 2015). Context cues are dynamic over people, time and space, and might be particularly important when facial affect cues are ambiguous or the emotion is nuanced and complex (Aviezer et al., 2017).

When context effects have been studied, stimuli have been relatively primitive, such as superimposing a face on an unrelated complex scene (de Gelder et al., 2006), presenting an isolated face image after a written comment (Schwarz et al., 2013), or presenting a foreground face with other faces in the periphery that vary in gaze direction and affect (Mumenthaler and Sander, 2012). While these contextually embedded stimuli are an improvement over isolated faces, their ecological validity, and hence their clinical utility, is limited because artificially juxtaposed images are not something typically encountered in everyday life.

A second limitation of most tools is that response choices are derivatives of the stereotyped “basic” emotions proposed by Woodworth almost a century ago (Woodworth, 1938), i.e., happy, sad, afraid, surprised, disgusted, and angry. These basic emotions were popularized in research by Ekman and others, beginning in the 1960s (Ekman and Friesen, 1967). Ekman and colleagues argued that the basic emotions were “universal” (Ekman et al., 1969; Ekman, 1992), and thus should be used in emotion recognition research (Ekman et al., 1972). The Ekman and Friessen black-and-white photographs were the gold standard stimuli for decades of research, and basic emotions continue to dominate experimental stimulus sets. Studies using basic emotion stimuli have been fruitful, as results have shown impairments in many neurological populations, including TBI (Schmidt et al., 2010; Babbage et al., 2011), as well as multiple sclerosis (Henry et al., 2009; Charvet et al., 2014), brain tumors (Mu et al., 2012), stroke (Yuvaraj et al., 2013), Parkinson disease (Heller et al., 2014), Huntington’s Disease (Kipps et al., 2007), frontotemporal dementia (Kumfor and Piguet, 2012), and alcohol use disorders (Pabst et al., 2022). It has been increasingly recognized, however, that basic emotions represent a fraction of felt and displayed emotions in everyday life (Fernández-Dols and Crivelli, 2013, 2015; Aviezer et al., 2017). Despite evidence of their construct limitations, basic emotion categories continue to dominate clinical and experimental stimuli, even when using new technology such as 3D imaging (Lott et al., 2022) and virtual reality (Geraets et al., 2021).

One category of emotions that is common in everyday life but typically not included in test stimuli is social emotions. Social emotions can be defined as emotions that are interpretable only in the context of social information, including information in the social context and interactions with others, as well as inferences about others’ mental states (Buck, 1988; Adolphs, 2002). Social emotions can be negative, such as grief and despair, or positive, such as admiration and pride (Tamietto et al., 2007). As with basic emotions, there is strong evidence that recognition of social emotions is impaired in many neurological populations, including TBI (Turkstra et al., 2018), schizophrenia (Bora et al., 2006), epilepsy (Broicher et al., 2012), multiple sclerosis (Charvet et al., 2014), and Huntington’s Disease (Eddy et al., 2012). By definition, social emotions are understood in a social context, so stimuli to test recognition of these emotions should include features of social context.

The third limitation of most assessment tools is that the response modality is forced choice, where participants are asked to select the word that best describes the emotion shown by a person in a photograph or video. Choices typically are the same basic emotions listed above or a mix of basic and social emotions. A historical example of the latter is the Reading the Mind in the Eyes test (Baron-Cohen et al., 2001a), which includes variants of basic emotion words (e.g., sad vs. despondent, angry vs. annoyed, afraid vs. terrified) and social emotion words (e.g., disappointed, jealous), and also cognitive state terms (e.g., bored, preoccupied), mental action terms (e.g., fantasizing), personality characteristics (e.g., shy, decisive), and judgments about the person rather than what they are feeling (e.g., arrogant, friendly). Several studies have shown evidence of impairments on forced-choice like the Eyes Test in adults with TBI (e.g., Havet-Thomassin et al., 2006; Turkstra, 2008; Muller et al., 2009; Ubukata et al., 2014; Rodriguez-Rajo et al., 2022), but it is not clear that the tools assess what respondents think vs. how well they can map their thoughts to response choices.

Zupan et al. (2022) argued that forced-choice tasks using only basic emotions are unlikely to “capture nuances in how people think about and perceive emotion” (p. 3). Their argument was based on the potential mismatch between an individual’s emotional lexicon, which is likely to be idiosyncratic, and the standard response options on emotion recognition tasks. As an illustration, Zupan et al. asked typical adults to label emotions using an open-ended response format, then asked novel raters to categorize those responses according to the basic emotion categories happy, sad, angry, fearful, and neutral. Degree-of-fit ratings were calculated for each word, based on the frequency with which raters assigned that word to each category. Results showed that other than happy, which was the only positively valenced word, the degree of fit was low across categories, i.e., the words people generated in the open-labeling task did not fit neatly into the basic emotion categories.

In a precursor to the present study, Turkstra et al. (2017) presented photographs of faces in isolation or in real-life scenes to a sample of university students, and asked participants what they thought the person in the photograph was feeling. Only 28% of responses were basic emotion terms. More than one third of those were “happy” or “happiness,” consistent with the findings of Zupan et al. (2022) and other studies showing happy is the easiest emotion to label (Rosenberg et al., 2014; Hayes et al., 2020). Other studies have likewise shown content differences between open- vs. forced-choice affect labeling in typical children (e.g., Cassels and Birch, 2014) and adults (e.g., Winters, 2005). These results suggest that forced-choice formats test an individual’s ability to identify which response option is most like what they think the person in the photograph is feeling, rather than what they actually think the person is feeling. This limitation could be particularly problematic for individuals with TBI, who are known to have challenges with inference (Johnson and Turkstra, 2012) and decision making (Bonatti et al., 2008).

Cassels and Birch (2014) compared children’s responses on open- vs. forced-choice versions of the Eyes Test described above (Baron-Cohen et al., 2001b). Eyes Test scores were higher for the closed-ended than open-ended version, but scores on the latter had higher correlations with constructs like empathy that the Eyes Test should measure. The open-ended version was more sensitive to group differences between children with and without learning disabilities, and had a lower correlation with vocabulary test scores, i.e., scores were not confounded by vocabulary ability. Minimizing vocabulary confounds is particularly important in TBI research, given the verbal recall challenges often associated with TBI.

In summary, while existing emotion recognition tasks have shown differences between adults with and without TBI, characteristics of task structure limit our understanding of how people with TBI identify emotions “in the wild.” To address limitations of existing tasks, we created an open-labeling task using complex visual scenes from real-life photographs, and asked adults with and without TBI to state what the person in each photograph was feeling. Based on previous studies in TBI, we expected group differences in the content of emotion labels. As the images were from real-life situations, we also expected that both groups would use proportionately fewer basic emotion words and more social emotion and other words (e.g., cognitive-state terms and evaluative terms). For insight into any group differences, a subset of participants completed the task with eye-tracking equipment, so we could analyze where participants looked in the images. We hypothesized that if verbal responses of adults with TBI differed qualitatively from those of adults without TBI, that difference might be attributable to TBI group participants looking at the different places in the image, specifically looking either longer or more frequently at the face than at the context in which that face was situated.

## 2. Materials and methods

### 2.1. Participants

Participants were 55 adults (29 females) with moderate–severe TBI and a comparison group of 55 uninjured adults, matched individually for age ± 5 years, race, and sex. If the participant reported that their TBI occurred after they completed their formal education, they were matched for years of education. If the injury occurred when the participant with TBI was in school, participants were matched on educational trajectory, operationalized as typical grades in school and intentions to pursue further schooling. All participants were recruited from the Midwestern United States as part of a larger study of social cognition in adults with TBI.

TBI severity was defined according to standard injury criteria (Malec et al., 2007), i.e., a loss of consciousness of 30 min or more or post-traumatic amnesia of 24 h or more, or a lowest Glasgow Coma Scale (Teasdale and Jennett, 1974) score of less than 13 in the first 24 h; and evidence of cortical or brainstem damage. Other inclusion criteria were self-identification as a Native English speaker and no reported history of a diagnosis of language or learning disability or neurological disorder affecting the brain, other than the TBI. Exclusion criteria were failing a pure-tone hearing screening test at 20 dB HL at 500, 1,000, 2,000, and 4,000 Hz; failing standard screenings for far and near vision; or testing in the aphasic range on the Western Aphasia Battery Bedside Screening Test (Kertesz, 2006).

### 2.2. Tasks

#### 2.2.1. Emotion-in-context task

**Figure 1:**
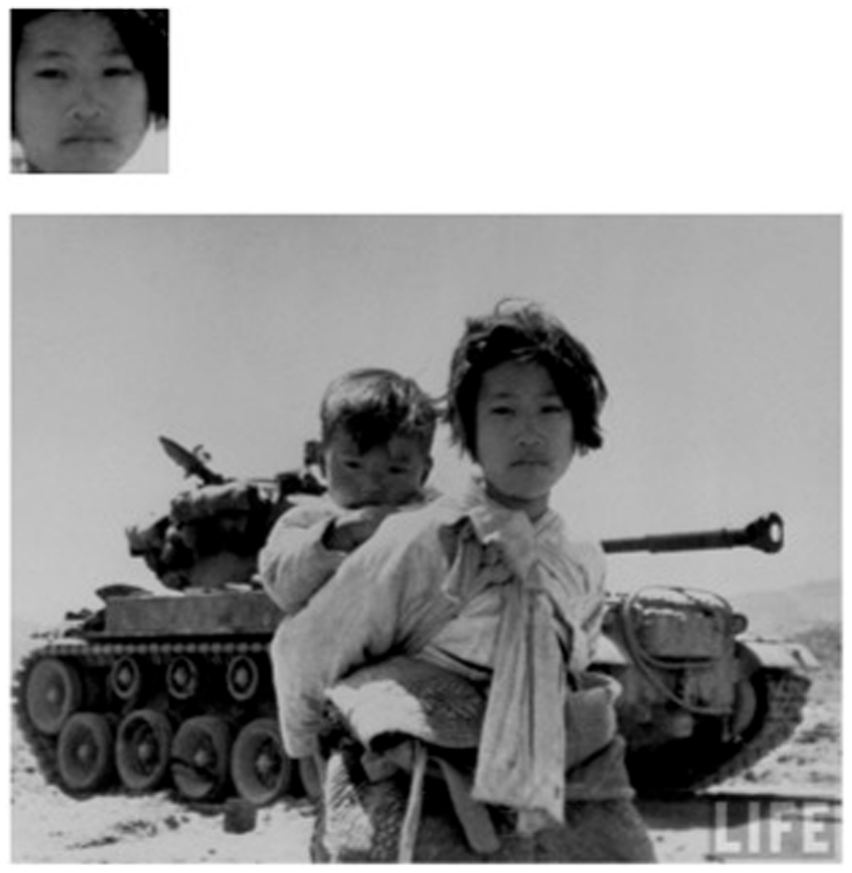
Sample stimuli with face only and face in context. Image source: Interim Archives / RV Spencer. Reproduced with permission from Getty Images.

The emotion-in-context (EIC) task was comprised of 60 photographs from the LIFE Magazine online archives, chosen because they appeared to be emotionally evocative, captured people in real-life scenarios, showed at least one individual with a clearly visible facial affect display, had visual contexts that could influence interpretation of affective displays, and appeared to depict a range of basic and social emotions (emotion types were not determined *a priori*, as that was the goal of the study). Each photograph was presented in one of two formats: as a full photograph in its original form, with a one-inch square box drawn around the face to be labeled (face-in-context items, FC), and as a cropped image of only the face (face only, FO). Fifty-seven of the 60 faces (boxed in the FC condition or alone in the FO condition) were sized at one-to-two inches per side. Three images of faces alone had one or two sides that were three inches, a technical error that will be discussed in the limitations. Sample FC and FO stimuli are shown in Figure 1. Photograph order was randomized then fixed, so that each photograph was randomly assigned to appear in either the FC or FO condition.

The EIC task was administered individually via a laptop computer, in a quiet room. Faces in the FO condition and boxed faces in the FC condition were centered on the screen (i.e., scenes were displayed so that the boxed face was at the center of the screen). Participants were seated approximately 16 inches from the laptop display and fitted with the SMI eye-tracking glasses (2012). The table was fitted with a chin-rest and height was adjusted to be comfortable for each participant. Each participant completed a standard calibration protocol, then began the task. For each image the participant was asked, “What emotion is this person feeling?” Spoken responses were written down verbatim by a research assistant seated out of sight of the participant. Participants completed the task at their own pace.

#### 2.2.2. Cognitive tests

To compare the present study to previous publications, participants completed a series of tasks recommended by the Common Data Elements Committee for TBI research (Wilde et al., 2010). These were the California Verbal Learning Test (CVLT) (Delis et al., 2000), Wechsler Adult Intelligence Scales Processing Speed Index tests (WAIS-PSI) (Wechsler, 2008), and Trail-Making Tests A and B (Tombaugh, 2004).

### 2.3. Procedure

Participants completed the tests to characterize cognitive functions in the first or second session of the larger study, in a randomized order. The EIC task was scheduled on the second day of testing, after a non-emotion task, to avoid priming of affective responses. Participants had, however, completed two forced-choice emotion recognition tasks in a previous testing session that occurred 1 day to two-and-a-half months prior to the EIC, depending on participant availability and scheduling constraints. Potential effects of that earlier testing will be considered in the Limitations section.

The relevant institutional review boards approved all procedures. The authors assert that all procedures contributing to this work comply with the ethical standards of the relevant national and institutional committees on human experimentation and with the Helsinki Declaration of 1975, as revised in 2008.

### 2.4. Analysis

#### 2.4.1. Verbal responses

Prior to analysis, all multiword responses were reduced to single words to eliminate any potential response length effects before analysis with Python’s natural language toolkit (Bird et al., 2009). Our reduction rule was to take the first label in each response (e.g., “angry, afraid” = angry), to avoid making mental state inferences about participants’ intents (e.g., that they were self-correcting).

#### 2.4.2. Sentiment analysis

We ran a sentiment intensity analysis using Python’s natural language toolkit (nltk) package to determine whether the valence and intensity of the emotion labels produced for each picture differed between participants in the TBI vs. comparison group. Nltk’s sentiment intensity analysis relies on the Valence-Aware Dictionary for Sentiment Reasoning model (Hutto and Gilbert, 2014), which uses a dictionary of lexical features and their corresponding human-rated emotional intensities to determine the sentiment of new text passages. For each input text passage, nltk outputs a “sentiment score” between −1 and 1, which is the normalized sum of the emotional intensities of all lexical features included in the input. Sentiment scores near −1 correspond to intense, negative sentiments, and sentiment scores near 1 correspond to intense, positive sentiments.

We hypothesized that the TBI group’s responses might differ from those of comparison participants in *both* valence directions—that is, we did not expect TBI responses to be only more positive or only more negative. To better understand how TBI responses differed, we calculated the “true” valence for each image (positive, negative, or neutral). These “true” valences were determined by two independent researchers individually rating their perceived valence for each image, and then comparing their ratings, ensuring they agreed on the valence for each image included. Disagreements were resolved by a third researcher.

We obtained sentiment scores for each participant’s response to each image and performed a linear mixed effects analysis predicting each participant’s sentiment score from their experimental group (TBI vs. control) using the lme4 package in R (Bates et al., 2014). Participants and images were entered into the model as random intercepts, and experimental group, true valence, and their interaction term were entered into the model as fixed effects. We included linear and quadratic contrasts for true valence in order to more clearly understand any directional differences we might find. Sex (male or female) was also entered into the model as a fixed effect, for an exploratory analysis based on mixed evidence of sex differences in emotion labeling (Turkstra et al., 2020). We noted that one image included in the original dataset was pixelated and difficult to see, potentially inhibiting their ability to see what emotion was depicted. Thus, participants’ responses to this image were removed from further analysis.

##### 2.4.2.1. Proportion of basic vs. social emotion words

We calculated the number of responses in the six basic emotion categories—happy, sad, disgusted, angry, afraid, and neutral—using wildcards to capture spelling and morphological variations (e.g., happy/happiness/happiness), and calculated percent of total responses for each word or spelling variant. We included either *afraid* or *fear* because affect labeling tasks commonly include either. To ensure that any group differences were not due to injury-related effects on word-finding in participants with TBI, we also calculated type-token ratio as a measure of lexical diversity in both groups. Data were summarized descriptively.

### 2.5. Gaze data

Gaze data were available for a subset of 37 participants: 18 in the TBI group (female = 9), and 19 in the comparison group (female = 12). For this analysis we were interested in whether participants looked at context cues, and thus used only the 30 FC stimuli. Trained research assistants coded the eye-tracking data from SMI Software (2012). For each fixation in each image, coders labeled the location of the fixation as Face (F), Scene (S), or Other (O), and calculated total fixation time and number of fixations for each area. Both total fixations and the number of fixations were calculated as both have been used as measures as attention.

Total fixation time and total number of fixations were compared between groups using a multivariate analysis of variance with TBI status, sex, and area of interest (face or scene) as independent variables and total number and duration of fixations as dependent variables.

## 3. Results

### 3.1. Demographics and cognitive test scores

Participant characteristics are shown in Table 1, including scores from cognitive tests recommended for TBI research (Wilde et al., 2010). Participants were predominantly Caucasian (*n* = 104), with 2 participants who self-identified as African American and 2 who self-identified as of mixed race. Analysis of variance (ANOVA) revealed a significant between-groups difference on all neuropsychological measures (*p*’s < 0.001). There were no significant sex-based differences on any measure except Trails A, and no significant interaction of group and sex (all *p*’s > 0.05). For Trails A, scores for women were significantly higher than scores for men, *F*(1, 109) = 6.71, *p* < 0.05. As there was no significant interaction of sex by group, this difference was not considered further in analysis.

**Table 1:** Demographic characteristics.

	Comparison group (*n* = 55)	TBI group (*n* = 55)
Age in years (range)	43.99 (19.33–72.33)	43.15 (21.08–75.75)
Males: Females	24:29	24:29
Years of education (range)	15.29 (12–19)	15.19 (12–23)
Years post-TBI (range)	n/a	9.83 (1–42)
Trails A (SD)	0.65 (0.85)	−0.43 (1.45)
Trails B (SD)	0.79 (1.12)	−1.33 (3.93)
WAIS-PSI (SD)	108.13 (21.83)	93.07 (17.85)
CVLT immediate T score (SD)	58.34 (8.0)	46.04 (12.27)
CVLT short delay Z-score (SD)	0.58 (0.89)	−0.52 (1.27)
CVLT long delay Z-score (SD)	0.60 (0.86)	−0.68 (1.43)

TBI, Traumatic Brain Injury; *M*, mean; SD, standard deviation; CVLT, California Verbal Learning Test (Delis et al., 2000); Trails A, Trail-Making Test Part A; Trails B, Trail-Making Test Part B (Tombaugh, 2004); WAIS PSI, Wechsler Adult Intelligence Scale (Wechsler, 2008; Abdallah, 2020) Processing Speed Index. Trails A and B scores are z-scores; CVLT, WAIS, and PSI scores are scaled scores.

### 3.2. Verbal responses

#### 3.2.1. Sentiment analysis

We found a significant main effect of true valence, *t*(75.1) = 14.78, *β* = 1.15, *p* < 0.001. This main effect was significant for the included linear contrast, suggesting that images with negative true valence were more likely to receive lower sentiment scores and images with positive true valence were more likely to receive higher sentiment scores, validating sentiment scores we calculated from participants’ responses.

**Figure 2:**
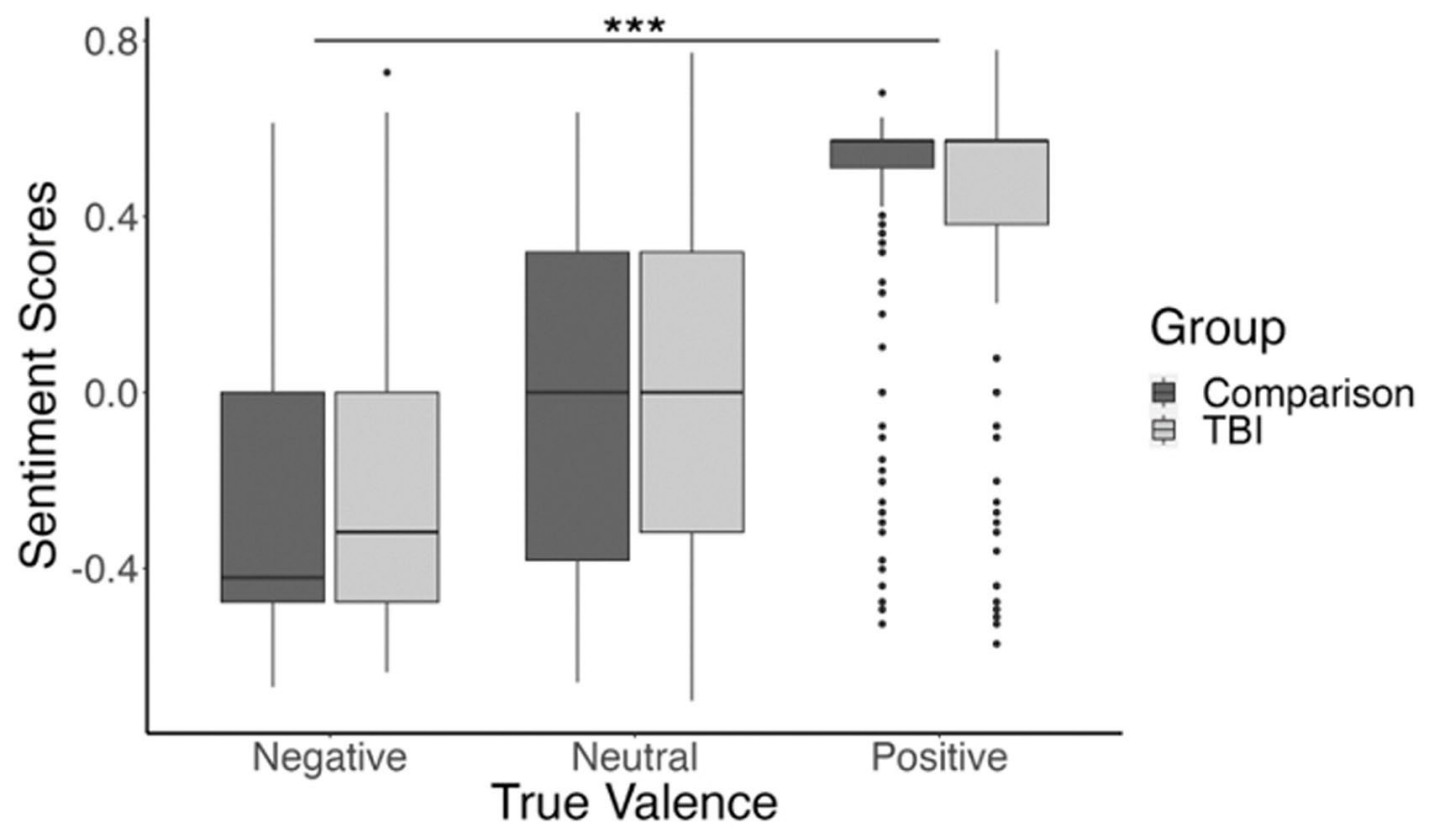
Boxplot showing participants’ mean sentiment scores for negatively valenced, neutral, and positively valenced images. Sentiment score means are shown in dark gray for the comparison group and light gray for the TBI group. ***p <0.001.

We also found a significant interaction between the TBI and comparison groups, and true valence (positive, neutral, or negative), *t*(5,641) = −4.37, *β* = −0.21, *p* < 0.001. This interaction was significant for the included linear contrast, indicating that the positive relationship between true valence and sentiment score was slightly *attenuated* for the TBI group compared to the comparison group. That is, TBI group responses to positive images were less positive than those of the comparison group, and their responses to negative images were less negative than those of the comparison group. The main effect of true valence, as well as the interaction between true valence and experimental group, are shown in Figure 2.

There were eight positively-valenced images with particularly high sentiment scores (*M* = 0.51, *SD* = 0.17). We were concerned that these images might be skewing the results in our initial analysis, so we completed a follow-up analysis with these images excluded. Results showed the same main effect of true valence, *t*(64.57) = 9.72, *β* = 1.1, *p* < 0.001; and interaction between true valence and group, *t*(4,852) = −3.82, *β* = −0.26, *p* < 0.001; suggesting that our effects were not driven by these images alone.

**Figure 3:**
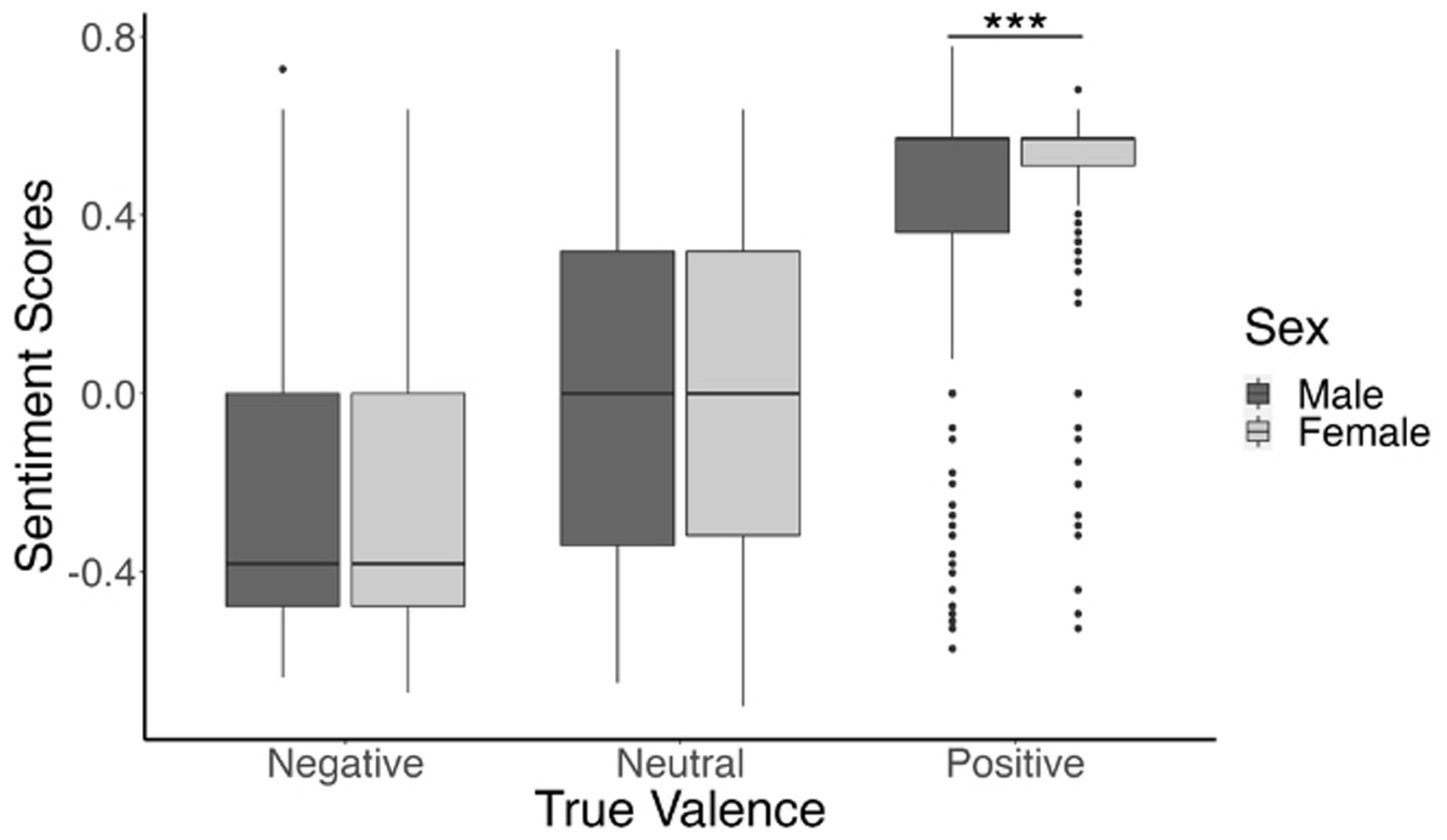
Boxplot depicting participants’ mean sentiment scores for negatively valenced, neutral, and positive images. Sentiment score means are shown in dark gray for males and light gray for females. ***p <0.001.

We also found a significant interaction between sex (male or female), and true valence (positive, neutral, or negative), *t*(5,639) = 2.81, *β* = 0.13, *p* = 0.005. This interaction was significant for the included linear contrast, indicating that the positive correlation between true valence and sentiment score was higher for the females compared to males. This higher correlation was primarily driven by female participants’ responses to positive images, and *post hoc* t-tests comparing males and females within each of the three true valence conditions confirmed this relationship [negative valence: *t*(2303.4) = 1.64, *p* = 0.1; neutral valence: *t*(1936.4) = −0.15, *p* = 0.88; positive valence: *t*(1310.4) = −4.49, *p* < 0.001]. These relationships are illustrated in Figure 3.

When reducing multi-word responses to single words for analysis, we observed that participants with TBI appeared to give more multi-word responses than the comparison group. The total number of multi-word responses in the TBI group was 258, median = 2, mode = 0, range = 0–30; and the total in the comparison group was 153, median = 1, mode = 0, range = 0–15. A median test showed this difference was not significant, chi2(1) = 3.05, Pr = 0.08. Two participants in the comparison group and seven in the TBI group generated more than 10 multi-word answers.

Three individuals in the TBI group also showed patterns that were not observed in the comparison group: one participant responded with “angry” for 22/60 items, vs. a range of 0–11 items for the remaining 99 participants (mode = 2); one participant said “serious” for 34/60 items and another participant said the same word for 14/60 items, vs. 0–10 for the remaining 98 participants (mode = 0); and one participant responded to 15 items with a description of what the person was doing (e.g., looking up at something, posing for a picture, waiting for someone to take a picture), a pattern that was not seen in any other participant in either group.

##### 3.2.1.1. Proportion of basic emotion words

The percent of possible responses for each group (*n* = 3,300 per group) in each of the five basic emotion categories is shown in Table 2. Overall, less than half of responses were basic emotion words. Type-token ratio was 0.15 in the comparison group and 0.20 in the TBI group, i.e., overall, participants with TBI generated more different words than their uninjured peers.

**Table 2:** Percent of possible responses in each group in the five basic emotion categories.

	Comparison group (*n* = 3,300)	TBI group (*n* = 3,300)
Happy	22.42	19.55
Angry	5.06	5.36
Sad	10.55	6.61
Disgusted	1.73	1.82
Neutral	5.00	2.97
Afraid/Fear	1.09	1.39
Total	45.85	37.70

#### 3.2.2. Gaze data

For the subset of participants with gaze data, there was a main effect of area of interest on number and total time of fixations, *F*(2, 65) = 11.28, *p* < 0.001; no significant effect of group, *F*(2, 65) = 1.28, *p* = 0.28, or sex, *F*(2, 65) = 0.50, *p* = 0.61; and no significant interaction of group by area of interest, *F*(2, 71) = 0.41, *p* = 0.66. Follow-up univariate tests showed a significant effect of area of interest on both number of fixations, *F*(1, 75) = 10.80, *p* < 0.005; and total fixation time, *F*(1, 75) = 23.70, *p* < 0.0001, with more fixations and time in the face region than the scene. Summary data for total number and duration of fixations are shown in Table 3. As there was no main effect of sex, data from male and female participants are combined in the table.

**Table 3:** Average total number and duration of fixations on the scene vs. face for FC stimuli (*n* = 30), for participants in the TBI and comparison groups.

	Comparison group		TBI group	
	Face	Scene	Face	Scene
Total number of fixations	146.53 (52.00)	110.26 (50.35)	145.32 (77.36)	93.79 (50.69)
Total fixation duration	39270.95 (15966.24)	21425.26 (10245.40)	44708.37 (32894.77)	19117.26 (9713.72)

SDs in parentheses.

## 4. Discussion

We showed photographs of faces alone or in real-life scenes to adults with or without TBI, and asked them to label what the pictured people were feeling. We chose an open-ended response format to capture what participants were thinking, rather than how well they could map what they were thinking to our researcher-generated categories of emotions. We predicted qualitative differences in verbal responses between adults with and without TBI, and that eye-tracking results would help explain those differences. Our predictions were partly supported: there were indeed qualitative differences in verbal responses between the two groups, but not in gaze patterns. We also found that fewer than half of responses were the “basic” six emotions traditionally tested in affect recognition studies, and about half of those were variants on the word “happy.” Our results add to the literature on affect recognition in adults with moderate or severe TBI, and raise questions about the ecological validity of forced-choice tasks for assessment of affect recognition after TBI.

### 4.1. Verbal responses

We hypothesized that adults with TBI would generate qualitatively different affect labels than their uninjured peers. Sentiment analysis of open-ended responses revealed that the valence of responses by adults with TBI was attenuated relative to that of their uninjured peers. For example, for one study image many comparison group responses were “confused,” “worried,” or “anxious.” For the same image, the most common response in the TBI group was “bored,” with rare responses of “curious” or even “interested.” It could be argued that the difference in valence was due to language limitations in the TBI group, i.e., that participants with TBI were less able to generate words for stronger emotions. This explanation is unlikely, as all participants passed a screening test for aphasia and participants were closely matched on education (or pre-injury educational trajectory if the injury occurred while the participant with TBI was in school), and perhaps most relevant, lexical diversity was actually higher in the TBI group than the comparison group. Also arguing against a lexical interpretation is what Cassels and Birch (2014) referred to as the “minimum verbal ability required to simply speak about emotions” (p. 16). Emotion labels tend to be high-frequency words, and among types of mental-state terms they are learned relatively early in life (Bretherton and Beeghly, 1982), thus it is unlikely that adults with TBI used attenuated words because they were unable to generate fewer words specifically for stronger emotions.

Attenuated valence is consistent with the emotional blunting and apathy that are commonly described in adults with moderate–severe TBI (Lane-Brown and Tate, 2011). Evidence includes a study using an online social game (Kelly et al., 2013), in which adults with TBI reported less hurt feelings than their uninjured peers when they were ostracized by other players. Similarly, in a study using evocative film clips (de Sousa et al., 2012), adults with TBI had less facial muscle activity, lower autonomic arousal, and less self-reported empathy than their uninjured peers. Emotional blunting has been linked to deficits in functions such as moral reasoning (Martins et al., 2012), and can have profound effects on family functioning (Worthington and Wood, 2018), so it merits further study. Not all adults with TBI show this reduction in intensity of feelings (Croker and McDonald, 2005; Amorapanth et al., 2018), however, and emotional blunting is only one component of the complex construct of apathy (Arnould et al., 2013; Quang et al., 2022), so future studies should consider individual differences and how detecting others’ feelings manifests in interpersonal interactions.

We hypothesized that because stimuli included complex visual scenes, participants in both groups would use fewer basic emotion words than social emotion or other words. This hypothesis was supported, as fewer than 50% of verbal responses in both groups were the traditional basic emotion words, and when *happy* responses were excluded, the number was less than 25%. The frequency of basic emotion words was higher than the previous study of young adults using this task (Turkstra et al., 2017), in which 28% of all responses were from the six basic emotion categories. One possible explanation, which we realized post-hoc, is that some participants had completed a force-choice emotion recognition task on the same day (i.e., their separate sessions were combined), and that task had the six basic emotion categories. Previous exposure to the six emotions could have biased participants’ responses toward the basic emotion categories. This bias likely explained some findings, such as several participants’ responses of “neutral,” a relatively low-frequency word for affect naming and one that is unlikely to be used by viewers naive to emotion recognition tasks. Effects of previous exposure also could explain why the percent of basic emotion responses was lower in the TBI group than the comparison group. Given their lower memory test scores, individuals with TBI might have been less likely than their peers to remember having seen the basic emotion words.

An unexpected finding was the number of times adults with TBI generated multiple words in response to an image. Although a median test showed no significant group difference, the number of multi-word answers was a gross measure and did not capture qualitative aspects of responses. Visual inspection of the data showed responses that could be classified as adding specificity or refining responses (e.g., “Waiting, attention directed,” “intense, focused”), changing the emotion (e.g., “Angry, afraid,” “Joking around, disgusted”), hesitating (e.g., “I do not know, hopeful”), and providing alternatives (e.g., “happy or satisfied”). It would be of interest to compare these qualitative features between groups. Based on evidence of impaired affect recognition in adults with TBI, participants in the TBI group might be more likely, for example, to give the wrong response first then self-correct. To avoid making inferences about participants’ intents (e.g., if they were self-correcting, refining answers, or disinhibited), we chose the first label they generated. Affect labeling must occur rapidly in everyday life, so the first response also was the most ecologically valid. Asking participants to explain their answers could provide further insights, with the caveat that this would be unnatural in everyday life. It also could be informative to look more closely at characteristics of participants who generated a large number of multi-word responses, and how these individual differences relate to other measures of social cognition, communication, and community outcome.

### 4.2. Gaze data

The second study hypothesis was that eye-tracking results would help explain any group differences between adults with and without TBI. That hypothesis was not supported. Participants in both groups looked more at the face, which is logical given that the task was to identify that person’s emotion, and there were no significant between-groups differences in total number or duration of fixations to the face vs. surrounding context. Two possible explanations for the discrepancy between verbal responses and gaze patterns are that (1) adults with TBI are looking at the same parts of the stimulus but perceiving or evaluating the information differently, or (2) adults with TBI are looking at different parts of the face, or fixating on parts of the stimulus in a different order than their uninjured peers (e.g., looking at the scene first rather than the face). The first explanation is consistent with the apathy studies cited earlier, and evidence of alexithymia after TBI (Fynn et al., 2021), and links between alexithymia and affect recognition (Neumann et al., 2014).

Results of a study by Greene et al. (2022) suggest the second explanation. These authors collected eye-tracking data while participants with TBI labeled static or dynamic images of facial expressions. They hypothesized that adults with TBI would have lower accuracy scores than their uninjured peers for both types of stimuli and would show “different eye scan patterns on static and dynamic tasks” (p. 3). Both the static and dynamic tasks had the typical basic emotion forced-choice options: anger, disgust, fear, surprise, happy, and sad (*sic*). Results replicated previous studies showing significant between-groups differences for recognizing anger, disgust, fear, and sadness; and no significant differences for happy, which as noted earlier is easiest for everyone, and surprise, which is most difficult for everyone (Jack et al., 2014). For both tasks, there was no main effect of group on fixation duration or number of fixations. The authors did find, however, differences in location of fixation on the static image task: participants with TBI looked first at the nose, which, as the authors stated, “is arguably the least informative part of the face” (p. 11), whereas controls looked first at the mouth. Greene et al. hypothesized that this finding was related to the lower accuracy scores in the TBI group but did not test that hypothesis directly. Fixation on regions within the face could be useful in future studies of context, as differences have been shown in populations with social similarities, such as adults with high-functioning autism (Setien-Ramos et al., 2022).

### 4.3. Limitations

A limitation of the study was that the use of news photographs was at the expense of experimental control. Photographs varied in image quality and in the number and nature of elements and people in each scene, so they are not directly comparable to each other or to other stimulus sets. As noted in the methods, for example, three of the FO images were sized differently than the others, and data for one image was discarded because the image was too pixelated to be labeled meaningfully. Future studies could use experimenter-generated images of scenes, to control factors such as exposure, focal length, and spatial frequency.

A second limitation of the study was that while the stimuli represented people of a wide variety of races and ethnicities, in a wide range of social, cultural, and economic contexts, the participants were almost all white European-heritage adults from the US Midwest. We employed several strategies to increase diversity of the sample but were unsuccessful. Literature on race and ethnicity effects has mostly focused on the race of the pictured person rather than the participant, other than the “in-group” effect (i.e., people are more accurate at identifying emotions on faces that look like their own race or ethnicity) (Elfenbein and Ambady, 2002; Dailey et al., 2010), and the TBI and comparison groups were matched, so it is not clear how the participants’ race would affect the study hypotheses. However, TBI occurs in all racial and ethnic communities, and studies that represent the TBI population are critical.

A third limitation is that for technical reasons, eye-tracking data were only available for a subset of participants. These participants were similar demographically to the full sample, and the TBI and comparison groups were well matched. Nevertheless, it is possible that we missed participants with TBI who had atypical gaze patterns.

## 5. Conclusion

In studies of affect recognition using de-contextualized stimuli and forced-choice responses, adults with TBI typically perform less accurately than their uninjured peers. It is not clear, however, if those studies capture the type of automatic, context-sensitive affect recognition that occurs in everyday life, vs. reflect the ability of adults with TBI to perform under specific task constraints. To better capture everyday affect recognition, we created stimuli with rich context cues and presented them in an open-response format. Results revealed qualitative differences in affect labels between adults with and without TBI, which did not appear to be attributable to differences in gaze patterns.

## Data availability statement

The raw data supporting the conclusions of this article will be made available by the authors, without undue reservation.

## Ethics statement

The studies involving humans were approved by the University of Wisconsin-Madison Social and Behavioral Science Institutional Review Board, University of Iowa Institutional Review Board. The studies were conducted in accordance with the local legislation and institutional requirements. The participants provided their written informed consent to participate in this study.

## Author contributions

LT was the senior researcher on this team, led much of the work cited, and led writing of the manuscript. SN created the original stimuli and co-developed the initial study concept with LT. BM and MD were co-investigators on the study and were responsible for developing the eye-tracking methods and oversaw implementation of all tasks and measures. SH-M updated the literature review, analyzed the gaze data, prepared the verbal response data for analysis, and contributed to manuscript preparation. SW analyzed the verbal response data and contributed to the manuscript. All authors contributed to the article and approved the submitted version.

## Funding

This work was supported by the NIH NICHD/NCMRR (award no. R01 HD071089) and NIH/NIGMS (award no. R25GMO83252).

## Conflict of interest

The authors declare that the research was conducted in the absence of any commercial or financial relationships that could be construed as a potential conflict of interest.

## Publisher’s note

All claims expressed in this article are solely those of the authors and do not necessarily represent those of their affiliated organizations, or those of the publisher, the editors and the reviewers. Any product that may be evaluated in this article, or claim that may be made by its manufacturer, is not guaranteed or endorsed by the publisher.
